# Integrated Analysis of Ulcerative Colitis Revealed an Association between *PHLPP2* and Immune Infiltration

**DOI:** 10.1155/2022/4983471

**Published:** 2022-03-16

**Authors:** Ting Li, Weidong Liu, Wenjia Hui, Tian Shi, Huan Liu, Yan Feng, Feng Gao

**Affiliations:** ^1^Xinjiang Medical University, Urumqi, Xinjiang, China; ^2^Department of Gastroenterology, People's Hospital of Xinjiang Uygur Autonomous Region, Urumqi, Xinjiang, China; ^3^Xinjiang Clinical Research Center for Digestive Diseases, China

## Abstract

Ulcerative colitis (UC) is a progressive intestine inflammatory disease that is prone to recur. Herein, we utilize microarray technology and bioinformatics to reveal the underlying pathogenesis of UC and provide novel markers. Colonic biopsies were taken from eight UC patients and eight healthy controls. Three differentially expressed miRNAs (DEMIs) and 264 differentially expressed genes (DEGs) were screened using mRNA and miRNA microarray. Most DEGs were significantly associated with immune response and were markedly enriched in the IL-17 signaling pathway. Among the target genes of DEMIs, PHLPP2 overlapped with DEGs and the downregulation of PHLPP2 group was mainly involved in the epithelial–mesenchymal transition. PHLPP2 was downregulated in UC patients, which was validated in 5 GEO datasets and qRT-PCR. The ROC curve demonstrated that PHLPP2 has a perfect ability to distinguish UC patients from healthy controls. Moreover, PHLPP2 was low expression in patients with active UC. CIBERSORT algorithm indicated that the abundance of gamma delta T cells (*P* = 0.04), M0 macrophages (*P* = 0.01), and activated mast cells (*P* < 0.01) was significantly greater than that of the control group. The Spearman correlation analysis showed that PHLPP2 was positively correlated with the proportion of activated NK cells (rho = 0.62, *P* = 0.013) and Tregs (rho = 0.55, *P* = 0.03), but negatively correlated with those of activated mast cells (rho = −0.8, *P* < 0.01) and macrophages (rho = −0.73, *P* < 0.01). These results indicate that PHLPP2 is associated with immune cells in the pathogenesis of UC, as well as provide new prospects and future directions of investigation.

## 1. Introduction

Ulcerative colitis (UC), a progressive and chronic inflammatory disease of the intestine, is one of the common inflammatory bowel diseases (IBDs) that cause huge burden on individuals and their family [1]. Genetic, environmental, and immunological factors contribute to UC pathogenesis [2, 3]. Despite major advances in therapeutic resources, the mechanisms underlying UC pathogenesis are multifaceted and have not been fully elucidated. Even with the newest treatments, remission rates are less than 50% and the emergence of drug resistance is inevitable [4]. Therefore, a comprehensive analysis of the pathogenesis and development of possible treatment strategies for UC are urgently required.

MicroRNAs (miRNAs) are a class of endogenous noncoding RNAs having a length of 19−25 nucleotides. miRNAs repress translation and promote mRNA degradation by interacting with the 3′ untranslated region of the target mRNA [5]. miRNAs are implicated in various pathological and physiological processes, such as cell proliferation, differentiation, apoptosis, and metabolism [6]. Recent studies have reported that miRNAs participate in the regulation of the intestinal mucosal barrier and mucosal immune system [7–9]. The aberrant expression of miRNAs has implications in UC development. A study of miRNAs can help explore the disease pathogenesis and identify new biomarkers.

The intestinal immune system is involved in UC progression [10]. During acute inflammation, patients present with heavy infiltration of inflammatory cells, including mast cells, lymphocytes, macrophages, and dendritic cells, in the intestinal mucosa [11]. Recent evidence suggests that some miRNAs and mRNAs can regulate the cytokine gene expression in immune cells and have essential roles in regulating the immune response. MiR-29 downregulates the interleukin- (IL-) 12/23 expression or indirectly downregulates the IL-23 expression by inhibiting activated transcription factor 2, thereby inhibiting the activation of Th17 cells [12]. Therefore, the evaluation of different types of infiltrating immune cells is important for elucidating the underlying mechanisms of UC pathogenesis.

In the present study, crucial genes and miRNAs associated with UC pathogenesis were identified using the microarray technique, and their expression, function, and interaction were evaluated using bioinformatics analyses. We also validated key genes in multiple independent datasets. Furthermore, we investigated the association between the identified biomarker and immune cell infiltration in UC.

## 2. Materials and Methods

### 2.1. Patients and Samples

The study was approved by the Ethics Committee of People's Hospital of Xinjiang Uygur Autonomous Region (No. 2015110). The diagnosis of ulcerative colitis was established on the basis of clinical, endoscopic, and histological criteria. Eight patients with UC and eight age-, gender-, and ethnically matched normal controls (NC) were enrolled. Normal controls with infectious diseases, autoimmune disease, or cancers were excluded. A written informed consent was obtained from all the study participants. Colonic biopsies were collected through colonoscopy. In patients with UC, biopsies were performed at the sites of active inflammation in rectum. The biopsy tissue was derived from the rectum in normal controls. Tissues were snap frozen in liquid nitrogen and stored at −80°C. Total RNA was isolated using the mirVana™ isolation kit. mRNAs and miRNAs were screened using Affymetrix PrimeView™ Human Gene Expression Array and Agilent Human miRNA Microarray (Release 21.0, 8 × 60K), respectively. Data analysis was performed using Agilent GeneSpring software.

### 2.2. Data Processing

Differentially expressed miRNAs (DEMIs) and differentially expressed genes (DEGs) between UC and normal tissues were identified using the “limma” package of R software. For the selection of DEMIs and DEGs, ∣log_2_ fold change (FC) | >1 was considered as the standard, and a *P* value of <0.05 was considered statistically significant. The DEGs and DEMIs were plotted using the “ggplot2” and “pheatmap” packages in R version 4.1.2.

### 2.3. GO, KEGG, and DO Analysis

To detect related signaling pathways and potential biological functions of the DEGs, gene ontology (GO) functional enrichment, KEGG pathway, and disease ontology analyses were performed using the “clusterProfiler” [13] and “DOSE” [14] R package. *P* value of <0.05 was considered as the criterion for statistical significance.

### 2.4. Identification of the Key Genes

Databases, namely, miRTarBase (http://mirtarbase.cuhk.edu.cn/php/index.php), miRDB (http://mirdb.org/), and TargetScan (http://www.targetscan.org/vert_72/), were used to predict the target genes of the DEMIs. Moreover, the intersection of the predicted target genes of DEMIs obtained using three databases was considered as the final target genes. The genes overlapping between the DEGs and final target genes were defined as the key genes.

### 2.5. Gene Set Enrichment Analysis

Gene set enrichment analysis (GSEA) version 4.1.0 software was used to further analyze the potential function of the key genes with a permutation of 1000. The patients were categorized into “high” and “low” groups based on the median expression value of the key genes. Hallmark pathways were performed to determine the crucial functions of the key genes. A false discovery rate (FDR) < 0.25 and a nominal *P* value of <0.05 referred to the statistically significant difference.

### 2.6. Validation of the Key Genes

The expression of key genes was further validated in GSE48958 [15], GSE73661 [16], GSE75214 [17], GSE87473 [18], and GSE92415 datasets. Additionally, the PHLPP2 expression in active and inactive patients was validated. The receiver operating characteristic (ROC) curve was plotted to assess the ability of the selected genes to distinguish between UC patients and controls.

### 2.7. Evaluation of Immune Cell Infiltration

To compare immune cell abundance between UC patients and healthy samples, we used our dataset, which comprised eight UC samples and eight healthy control samples, for the assessment of tissue-infiltrated immune cells. The “CIBERSORT” algorithm was used to calculate the relative proportions of 22 types of infiltrating immune cells. Significant alterations in immune cells were identified using the Wilcoxon test at *P* < 0.05. Pearson correlation test was performed to investigate the correlation between the expression of key genes and relative immune cell abundance. A *P* value of <0.05 was considered statistically significant.

### 2.8. Quantitative RT-PCR

Total RNA was extracted from colonic tissues using Trizol (Invitrogen, USA) and quantified by a NanoDrop spectrophotometer (Thermo). cDNA was synthesized using a TIANScript RT kit (Tiangen, Beijing, China). Quantitative PCR amplification of cDNAs was performed on a LightCycler 480 Real-Time PCR System (Roche, USA). The mRNA level of genes was detected with SuperRealPreMix Plus kits (TIANGEN, Beijing, China). Expression levels of target genes were normalized to *β*-actin mRNA levels. The primers for the qRT-PCR are listed in Table 1.

## 3. Results

### 3.1. Screening of Differentially Expressed mRNAs and miRNAs

**Figure 1:**
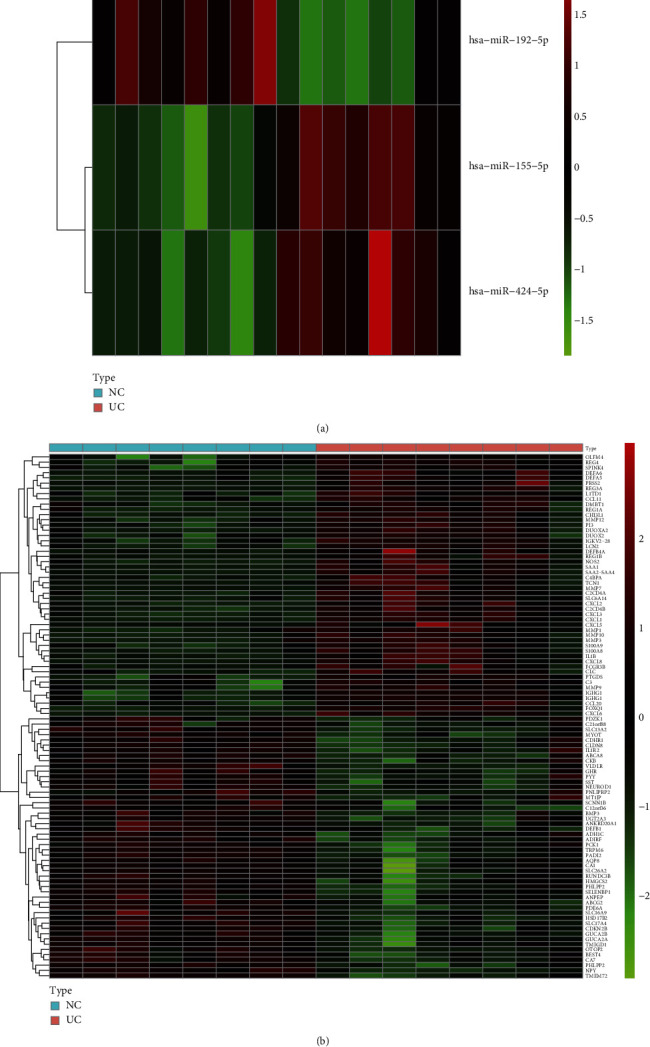
Identification of differentially expressed genes and differentially expressed miRNAs from mRNA and miRNA microarray. (a) Heat map of the DEMIs. (b) Heat map of the DEGs.

Table 2 presents the basic data of the participants in both groups. After analyzing with the criteria of adjusted *P* < 0.05 and ∣log_2_FC | >1, three DEMIs and 264 DEGs were screened. The expression of these genes and miRNAs is shown in Figure 1. For specific DEMIS and DEGs, see Supplementary Tables 1 and 2.

### 3.2. Functional Enrichment Annotation

**Figure 2:**
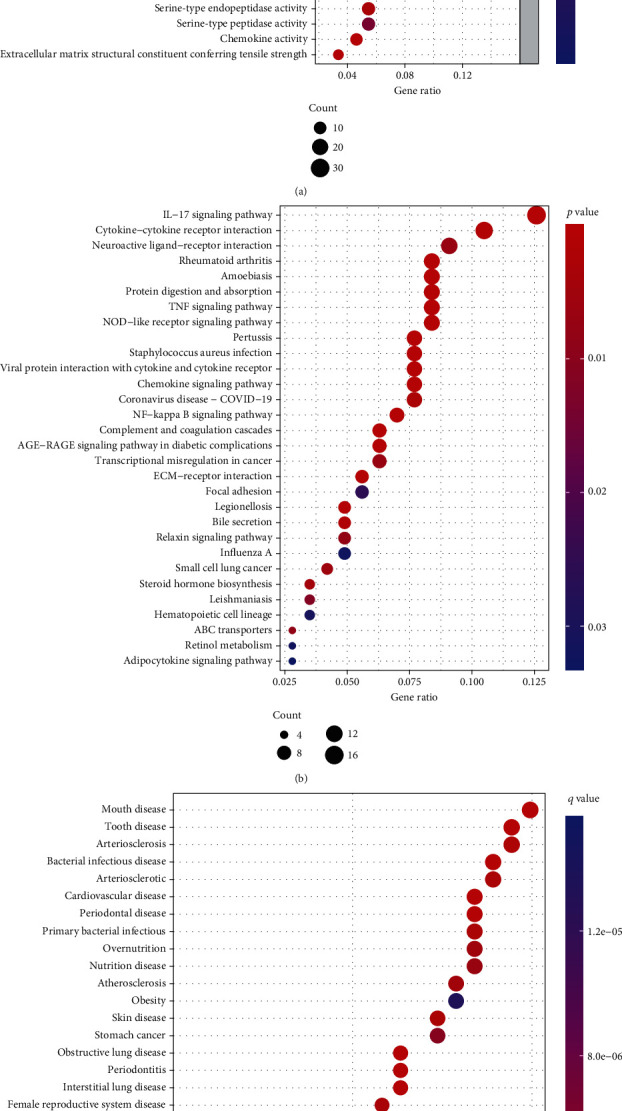
GO, KEGG, and DO analyses. (a) The bubble plot of enriched GO terms. (b) The bubble plot of the enriched KEGG pathways. (c) The bubble plot of the enriched DO pathways.

To further explore the functions of DEGs, we performed GO, KEGG, and DO analyses. The enriched GO annotation included immune response, extracellular matrix organization, and the regulation of inflammatory responses in the BP category. Endoplasmic reticulum lumen, collagen-containing extracellular matrix, and cytoplasmic vesicle lumen were included in the CC category. Receptor-ligand activity, endopeptidase activity, and extracellular matrix structural constituent were included in the MF category (Figure 2(a)). The KEGG pathway analysis mapped DEGs to the IL-17 signaling pathway and cytokine−cytokine receptor interaction pathway (Figure 2(b)). DO analysis suggested that these DEGs were closely linked with oral diseases (Figure 2(c)).

### 3.3. Identifying the Key Gene

**Figure 3:**
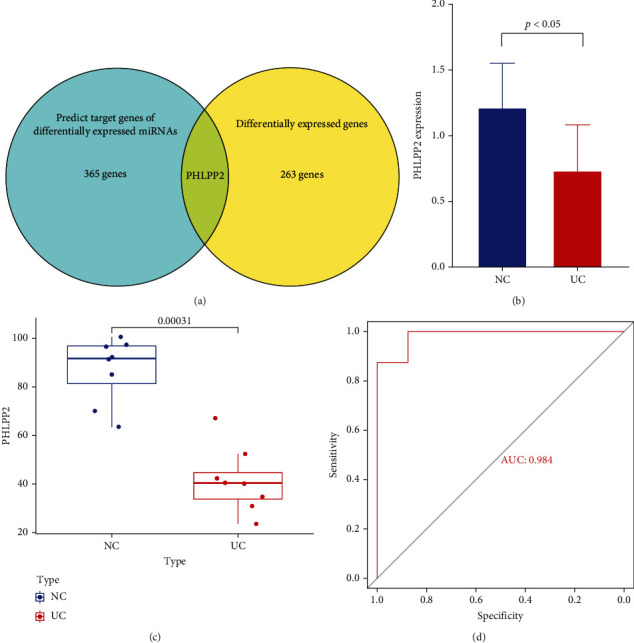
Identification of the key genes. (a) Venn diagram showing the overlap between differentially expressed genes and predicted targets of the differentially expressed miRNAs. (b) The qPCR results revealed that PHLPP2 expression levels were significantly different between UC and healthy controls. (c) PHLPP2 expression in the primary chip data. (d) ROC curve for PHLPP2 in the primary chip data.

Three databases (miRTarBase, TargetScan, and miRDB) were used to predict the target genes of the DEMIs. A total of 366 genes were obtained using the three databases and were considered as the final target genes. Only one gene, *PHLPP2*, intersected with DEGs (Figure 3(a)). The qPCR results revealed that PHLPP2 expression levels were significantly different between UC and healthy controls (Figure 3(b)). Analysis of the primary chip data showed that PHLPP2 was low expression in UC patients (Figure 3(c)). ROC curves indicated that PHLPP2 was a potential biomarker for distinguishing between UC patients and healthy controls in our patients' cohort (Figure 3(d)).

### 3.4. GSEA

**Figure 4:**
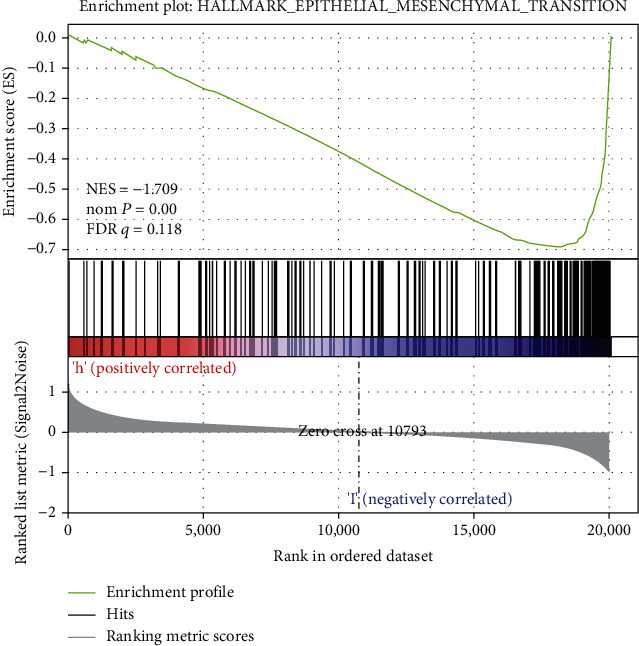
Gene set enrichment analysis (GSEA) showed PHLPP2 low expression group was mainly related to epithelial-mesenchymal transition.

GSEA is a computational approach for detecting minor undetectable changes in the gene expression. GSEA results indicated that the downregulation of the *PHLPP2* group was mainly involved in the epithelial–mesenchymal transition (Figure 4).

### 3.5. External Validation of the Key Genes

**Figure 5:**
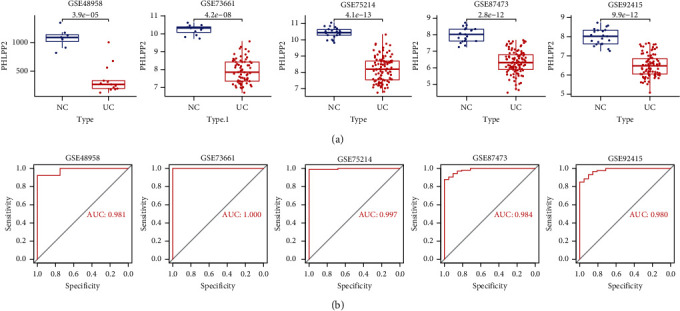
The expression level of PHLPP2 in the GEO database. (a) PHLPP2 was downregulated in UC patients in GSE48958, GSE73661, GSE75214, GSE87473, and GSE92415. (b) ROC curves reflecting the ability of PHLPP2 to distinguish between ulcerative colitis and healthy participants.

**Figure 6:**
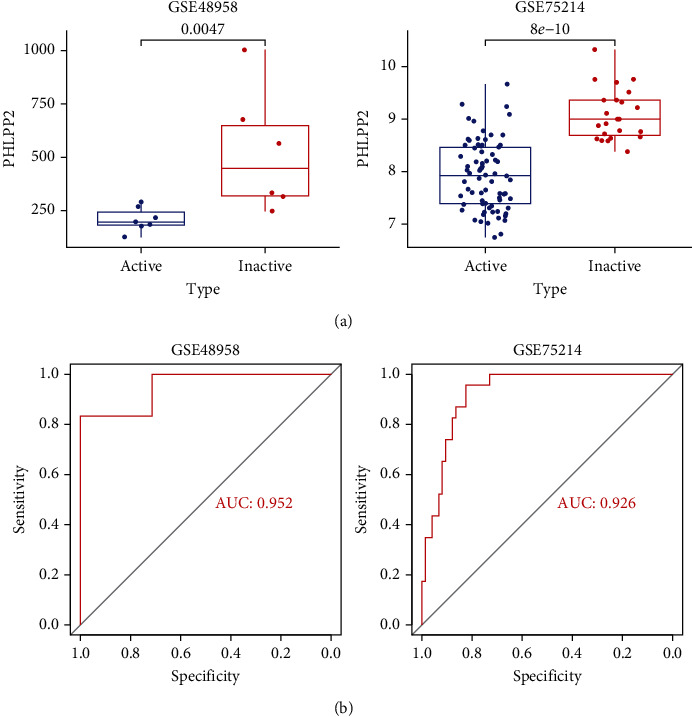
Validation of the expression of PHLPP2 in active and inactive UC patients. (a) PHLPP2 was low expression in patients with active UC. (b) ROC curves reflecting the ability of PHLPP2 to distinguish between active UC and inactive UC.

To further validate the ability of *PHLPP2* to distinguish between UC patients and controls, we examined the *PHLPP2* expression in GEO datasets. *PHLPP2* exhibited a significantly low expression in patients with UC from 5 datasets (GSE48958, GSE73661, GSE75214, GSE87473, and GSE92415) (Figure 5(a)). The ROC curve showed that *PHLPP2* has an ability to distinguish between UC patients and healthy controls (Figure 5(b)). We also validated the *PHLPP2* expression in active and inactive patients. The result shows that *PHLPP2* was low expression in patients with active UC (GSE48958 and GSE75214) (Figure 6).

### 3.6. Immune Cell Infiltration Analysis

**Figure 7:**
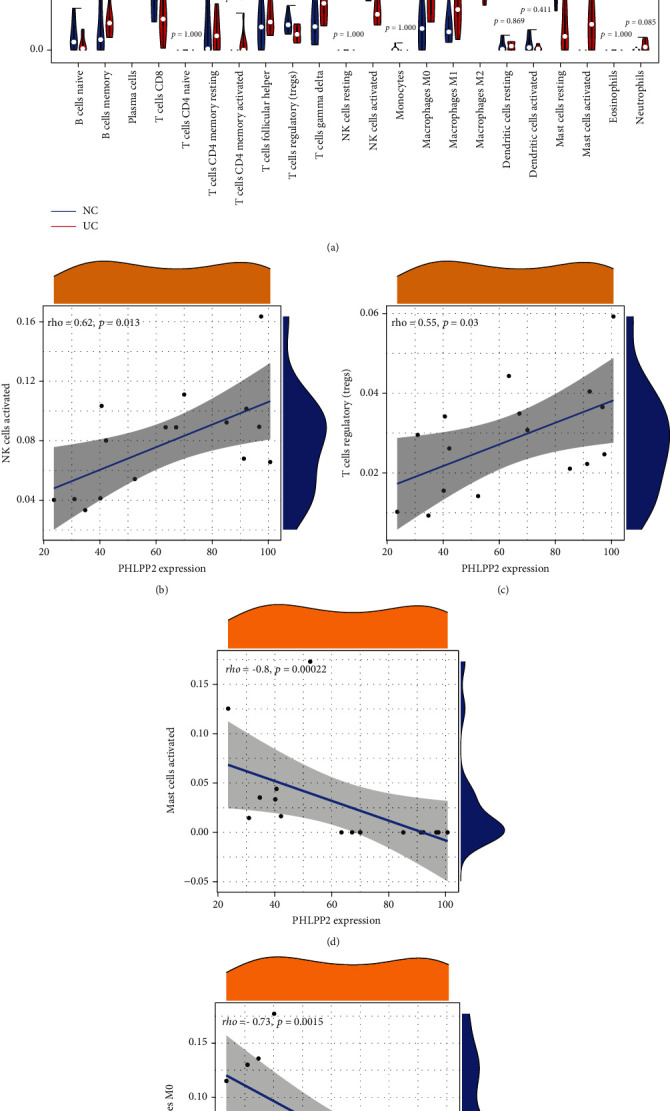
Evaluation of immune cell infiltration. (a) The proportion of 22 types of immune cells between normal samples and ulcerative colitis samples. The correlation analysis of PHLPP2 and the proportion of (b) NK cells, (c) Tregs, (d) mast cells, and (e) M0 macrophages.

In our data, the abundance of gamma delta T cells (*P* = 0.04), M0 macrophages (*P* = 0.01), and activated mast cells (*P* < 0.01) was significantly greater than that of the control group, whereas activated NK cells (*P* = 0.02) and resting mast cells (*P* < 0.01) showed the reverse expression pattern (Figure 7(a)). The Spearman correlation analysis of the expression of *PHLPP2* and immune cells showed that *PHLPP2* is positively correlated with the proportion of activated NK cells (rho = 0.62, *P* = 0.013) and Tregs (rho = 0.55, *P* = 0.03), but negatively correlated with those of activated mast cells (rho = −0.8, *P* < 0.01) and M0 macrophages (rho = −0.73, *P* < 0.01) (Figures 7(b)–7(d)).

## 4. Discussion

UC is an autoimmune disease with a long-lasting course and a high recurrence rate. Patients with long-standing and/or extensive UC have an increased risk of developing colorectal cancer. With the advent of next-generation sequencing and gene chip technologies, researchers can identify DEMIs and DEGs responsible for the initiation and progression of diseases. Therefore, combining the expression data derived from the microarray and comprehensive bioinformatics analysis is a convenient approach. In this study, three miRNAs (miR-424-5p, miR-155-5p, and miR-192-5p) and 264 mRNAs were screened. Among the predicted target genes of the DEMIs, *PHLPP2* intersected with DEGs. Therefore, we speculated that *PHLPP2* is associated with the pathological mechanism of UC.

Previous studies have reported the significance of the miRNAs, which were identified in our study. Studies have reported a high expression of miR-155 in activated UC patients. Compared with the control group, a 1.22- to 2.33-fold level change of miR-155 was increased in UC samples [19, 20]. miR-155 could participate in the regulation of UC development by negatively regulating the TLR4 signaling pathway via targeting SHIP1 and SCOS1 [21]. Another mechanism of miR-155 involved in UC would be to modulate the Th17 cell differentiation and antigen presentation in dendritic cells [22]. Our results are consistent with those reported by Zahm et al. and Wu et al. who found decreased expression of miR-192 in the sigmoid colon of patients with UC [23, 24]. However, the specific mechanism of action of miR-192 is unclear. The impact of miR-424 on UC is indistinctive. miR-424 has been studied mostly in tumors and is aberrantly expressed in multiple cancers such as renal clear cell carcinomas [25], colon cancer [26], chronic leukemia [27], pancreatic cancer [28], and ovarian cancer [29]. Moreover, miR-424 plays a dual role as it acts both as a tumor suppressor [30, 31] and as a cancer promoter [28, 32]. In conclusion, microRNAs are implicated in intestinal inflammation and immunity in UC, providing a new direction for exploring the pathogenesis and target-specific therapy for UC.

*PHLPP2* codes for homologous pleckstrin-homology-domain leucine-rich-repeat protein phosphatases. *PHLPP2* expression is considerably downregulated in various human cancers [33–36]. It can negatively regulate AKT and PKC signaling pathways by directly dephosphorylating AKT and PKC [37, 38]. Similarly, our data showed that *PHLPP2* is downregulated in UC patients, and this finding can be verified from multiple GEO datasets. We compared *PHLPP2* expression in active and inactive UC patients from the datasets and found that *PHLPP2* is downregulated in patients with active UC compared with that in patients with inactive UC. These results corroborate the findings of Wen et al. [39] who found that *PHLPP* downregulation led to an increase in the Akt activity with the consequently reduced intestinal epithelial cell (IEC) apoptosis. The short-term downregulation of *PHLPP* showed positive impacts on protection against excessive death of IECs. However, long-lasting activation of PI3K/Akt signaling induces IEC proliferation [40, 41], which ultimately results in the development of colitis-associated cancer [42]. Recent studies have also found that the loss of PHLPP2 expression contributes to epithelial pyroptosis facilitating rapid progression of colitis [43]. Additionally, using GSEA, we found that the low-expressed *PHLPP2* group was significantly enriched in the epithelial–mesenchymal transition- (EMT-) related pathway. As others have reported, *PHLPP2* knockdown induces an increase in the levels of EMT markers [44]. Intestinal wall fibrosis is a common late complication of UC characterized by superficial inflammation of the rectum and colon, and its intestinal fibrosis is generally limited to the submucosa [45]. Increasing evidence has supported a role for EMT in the pathogenesis of IBD-associated intestinal fibrosis and potentially suggests the use of the EMT signature as a molecular tool to assess cancer risk in patients with active UC lesions [46, 47]. Taken together, we can infer that chronic and repeated inflammation-induced PHLPP loss contributes to the development of colitis-associated cancer.

Results of the KEGG analysis indicated that DEGs were mainly enriched in the IL-17 pathway. IL-17 is an important proinflammatory factor that promotes the infiltration of inflammatory cells in UC [48]. Genetic polymorphisms in the IL-23/IL-17 axis have a substantial impact on UC [49]. Several IL-17-targeted therapeutic agents have been developed and proven effective in most animal experiments in IBD in recent years [50]. The IL-17-targeted therapy may become a potential new therapy for UC in the future.

Innate and adaptive immunity are the two dominant factors that drive the progressive tissue damage in patients with UC [51]. Our results suggested that *PHLPP2* is positively correlated with the proportion of activated NK cells and Tregs but negatively correlated with activated mast cells and M0 macrophages. CD4^+^, CD25^+^Tregs have immunosuppressive effects on the UC pathogenesis and have a major contribution in maintaining the intestinal immune homeostasis [52]. Tregs secrete IL-10 and TGF-*β*, which protect against colitis by suppressing the immune response [53]. A decrease in Tregs is related to UC pathogenesis [54], which can be because of the reduction in the quantity and function of Tregs, leading to the differentiation of CD4^+^ T cells to Th17 cells. The increased proportion of Th17 cells further promotes the secretion of inflammatory cytokines, which contribute to the progression of inflammatory responses [55]. Previous studies have reported that the proportion of macrophages increased with time in the mucosal, submucosal, and muscular layers of rats treated with 2,4,6-trinitrobenzene sulfonic acid and dextran sulfate sodium [56, 57]. Patients with UC exhibit severe infiltration and macrophage accumulation in the lamina propria of the colonic mucosa, which result in hypersensitivity to the stimulation by bacteria and their products and generation of abundant proinflammatory cytokines [58]. Targeted regulation of the polarization state of macrophages is a potential target for UC remission. An increased number of mast cells were first identified by Nolte et al. in the colonic tissue of UC patients [59]. King et al. [60] subsequently found that during the active phase of UC, the number of mast cells was 6.3 times higher than its normal range in the inflammatory region, 19.5 times higher at the junction of diseased and normal tissue, and 15.8 times higher in the normal intestinal segment. Several studies have reported that mast cells have profound consequences in UC pathogenesis [61, 62]. Mast cells, after being stimulated by various factors, are activated and release various mediators and cytokines, which mediate the inflammatory response in the intestine [63, 64]. Therefore, the inhibition of the activation of enteric mast cells might be a promising therapy for UC [65]. Our results further indicated that *PHLPP2* functioned in UC by regulating the immune cells.

## 5. Conclusions

*PHLPP2* was identified as a candidate biomarker that may participate in UC pathogenesis. *PHLPP2* was found to be correlated with the infiltration of immune cells, particularly Tregs, macrophages, and mast cells. The biological functions and pathways of the selected genes offer a holistic understanding of the underlying molecular mechanisms in UC. However, a more detailed investigation is required to validate the proposed mechanism.

## Figures and Tables

**Table 1:** Primer sequences.

Name	Primer sequence
PHLPP2	F-CTTACATCTCGTCCTTTGCACT
	R-GGTCGTTCAGTAGGTTCCAGTC
*β*-Actin (human)	F-CATGTACGTTGCTATCCAGGC
	R-CTCCTTAATGTCACGCACGAT

**Table 2:** Clinical characteristics of the study population.

	Ulcerative colitis group	Healthy control group
Number of patients	8	8
Mean age in years (y) (±SD)	45.50 ± 11.58	45.63 ± 11.30
Male sex	50%	50%
Ethnicity		
Han	50%	50%
Uygur	50%	50%

## Data Availability

The datasets used in this study can be obtained from the corresponding author upon reasonable request. The public data source is Gene Expression Omnibus database with the accession GSE48958, GSE73661, GSE75214, GSE87473, and GSE92415 (https://www.ncbi.nlm.nih.gov/geo/).

## Supplementary Materials

Supplementary 1 Supplementary Table 1: differentially expressed miRNAs.

Supplementary 2 Supplementary Table 2: differentially expressed genes.
